# Связь между исходом лечения и возрастом у пациентов с туберкулезом и сахарным диабетом: популяционный анализ

**DOI:** 10.14341/probl13252

**Published:** 2023-11-12

**Authors:** Л. Т. Ералиева, А. М. Исаева

**Affiliations:** Национальный медицинский университет; Национальный научный центр фтизиопульмонологии РК; Национальный медицинский университет

**Keywords:** туберкулез, сахарный диабет, Казахстан, смертность, исход лечения

## Abstract

**ОБОСНОВАНИЕ:**

ОБОСНОВАНИЕ. Несомненная важность этой работы состоит в том, что впервые в Республике Казахстан проводится анализ связи между возрастом и исходом лечения у пациентов с коморбидным диагнозом «туберкулез и сахарный диабет».

**ЦЕЛЬ:**

ЦЕЛЬ. Выявление корреляции между возрастом пациентов и исходом лечения у пациентов с туберкулезом и сахарным диабетом.

**МАТЕРИАЛЫ И МЕТОДЫ:**

МАТЕРИАЛЫ И МЕТОДЫ. Кросс-секционное ретроспективное исследование 2125 пациентов с туберкулезом в сочетании с сахарным диабетом из общего количества (43 807) пациентов, у которых диагностирован туберкулез (2017–2019 гг.). В исследовании проанализированы данные пациентов с коморбидностью из всех регионов Казахстана (данные 14 областей и 3 городов республиканского значения) (2017–2019 гг.).

**РЕЗУЛЬТАТЫ:**

РЕЗУЛЬТАТЫ. Выявлена высокая распространенность заболеваемости туберкулезом с сопутствующим диагнозом сахарного диабета в возрастной категории от 45 до 64 лет. Данную группу составили 1193 больных из 2115 (56,4% от общего количества пациентов с туберкулезом и сахарным диабетом). Средний возраст всех исследуемых пациентов с СД составил 54,7±13,4 года. Отмечается положительная корреляция между возрастом и исходом лечения пациентов с туберкулезом. Смертность была выше у представителей возрастной группы старше 45 лет — OR (95% CI) 0,213 (0,019–2,362), p — 0,0000015 (p<0,05).

**ЗАКЛЮЧЕНИЕ:**

ЗАКЛЮЧЕНИЕ. В результате исследования за 2017–2019 гг. был получен материал, анализ которого позволил заключить, что исход лечения в виде смертности прямо пропорционален возрасту пациента. Т.е. у представителей старших возрастных групп вероятность смертности выше, чем у молодых пациентов.

## Abstract (en)

**Background:**

Background. The undoubted importance of this work lies in the fact that for the first time in the Republic of Kazakhstan, an analysis is being made of the relationship between age and treatment outcome in patients with a comorbid diagnosis of tuberculosis and diabetes mellitus.

**Purpose of the study:**

Purpose of the study. Identification of the correlation between the age of patients with tuberculosis with diabetes mellitus and the outcome of treatment.

**Materials and methods:**

Materials and methods. Cross-sectional retrospective study of 2,125 patients with TB and diabetes mellitus out of a total of 43,807 of all patients diagnosed with TB (2017-2019). The study analyzed the data of patients with comorbidity from all regions of Kazakhstan (data from 14 regions and 3 cities of republican significance) (2017-2019).

**Results:**

Results. A high prevalence of tuberculosis morbidity with a concomitant diagnosis of diabetes mellitus in the age group from 45 to 64 years was revealed. This group consisted of 1193 patients out of 2115 (56.4% of the total number of patients with tuberculosis and diabetes mellitus). The average age of all studied patients with DM was 54.7±13.4 years. There is a positive correlation between age and treatment outcome in TB patients. Mortality was higher in the age group over 45 years old – OR95%CI = 0.213 [0.019–2.362], p – 0.0000015 (p < 0.05).

## ОБОСНОВАНИЕ

Республика Казахстан (РК) является одной из стран Европейского региона ВОЗ, которая определила борьбу с туберкулезом (ТБ) в качестве ключевого приоритета здравоохранения, поскольку Казахстан входит в число 30 стран мира с самым высоким бременем ТБ с множественной лекарственной устойчивостью [1]. Индивидуальные риски, такие как возраст, пол, курение, сахарный диабет (СД), статус вируса иммунодефицита человека, пребывание в местах заключения и статус мигранта, были связаны с ТБ [2]. Также после распада Советского Союза нестабильная экономическая ситуация имела свое влияние на здоровье населения. В период с 2007 по 2016 гг. в Казахстане отмечалось устойчивое снижение частоты распространения ТБ на 6% в год.

Бремя СД растет во всем мире, и на 2020 г., по оценкам Международной федерации диабета (International diabetes federation), около 463 млн человек живут с диабетом, что примерно составляет около 8,9% от общей численности населения мира [3].

Считается, что только в Казахстане число больных диабетом превышает 300 000, и в это число входят только пациенты, которым врачи поставили прямой диагноз. При этом существуют серьезные проблемы из-за нехватки квалифицированных специалистов по диабету, а это означает, что диабет часто лечится на поздних, а не на ранних стадиях заболевания. Эти недостатки привели к увеличению числа больных СД в Казахстане, СД в настоящее время является четвертым по распространенности заболеванием в стране и представляет собой растущую проблему общественного здравоохранения, которая влияет не только на здоровье человека, но и на систему здравоохранения в целом, а также на мировую экономику [4].

Для людей с СД ТБ может считаться одним из важных факторов риска — пациенты с СД имеют в 2,5 раза более высокий риск заболеть ТБ [5–7]. Таким образом, коморбидность повышает процент рецидива и летальных исходов. Данную ситуацию сегодня в мире называют коэпидемией СД и ТБ.

## ЦЕЛЬ ИССЛЕДОВАНИЯ

Данная работа была направлена на выявление корреляции между возрастом и исходом лечения у пациентов с ТБ, имеющих СД как сопутствующее заболевание. Проведен анализ эпидемиологических данных за 3-летний период с 2017 гпо 2019 г. по следующим критериям: распределение по возрастной структуре, полу, месту жительства; локализации туберкулеза: легочный, внелегочный или сочетанный (легочный с внелегочным).

## МАТЕРИАЛЫ И МЕТОДЫ

## Место и время проведения исследования

Место проведения. РГП на ПХВ «Национальный научный центр фтизиопульмонологии РК» МЗ РК, г. Алматы, Республика Казахстан.

Время исследования. Период с 2017 по 2019 г.

## Изучаемые популяции (одна или несколько)

Популяция: одна.

Критерии включения: подтвержденный ТБ и сопутствующий диагноз СД.

Критерии исключения: отсутствие СД.

## Способ формирования выборки из изучаемой популяции (или нескольких выборок из нескольких изучаемых популяций)

Сплошной.

## Дизайн исследования

Одноцентровое одномоментное ретроспективное исследование.

## Методы

Использованы ретроспективные данные о пациентах из информационной системы «Национальный регистр больных туберкулезом (НРБТ)» за период с 2017 по 2019 г. Для проведения статистического анализа использовался кросс-секционный метод исследования.

В НРБТ в изучаемые годы состояли под наблюдением 43 807 пациентов с подтвержденным диагнозом ТБ. В базу входят данные из 14 областей и 3 городов республиканского значения. Основным фильтром для отбора было наличие сопутствующего диагноза СД. Всего было отмечено 2115 пациентов с коморбидностью СД и ТБ. Поэтапный метод исследования показан на рисунке 1.

**Figure:**
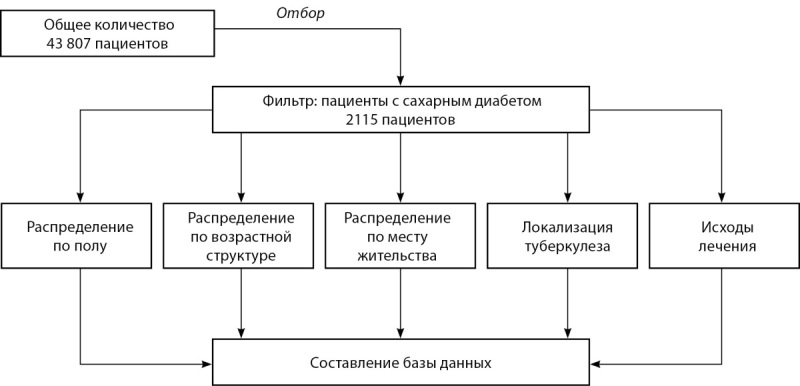
Рисунок 1. Пошаговый метод составления базы данных пациентов с коморбидным диагнозом для дальнейшего использования в статистических программных обеспечениях.

## Статистический анализ

Статистическая обработка данных проводилась с использованием программного обеспечения Statistica 13.3 и Microsoft Office Excel 2010. Статистическая значимость, корреляция переменных оценивались с помощью критерия Пирсона χ² (хи квадрат). Критический уровень значимости при проверке статистических гипотез — p<0,05.

## Этическая экспертиза

## ОЖИДАЕМЫЕ РЕЗУЛЬТАТЫ

За три года в РК отмечается снижение заболеваемости ТБ, при этом количество пациентов с СД растет с каждым годом (табл. 1).

**Table:** Таблица 1. Характеристика исследуемой когорты

Критерии оценки	Год
2017	2018	2019
Общее количество пациентов с ТБ	17 700	14 020	14 320
Количество пациентов с СД	708	694	713
Распределение по возрастной структуре
<14	2	0	1
15–24	15	10	11
25–44	144	132	126
45–64	396	401	396
>65	151	151	179
Распределение по полу
Мужчины	414	408	424
Женщины	294	286	289
Распределение по месту жительства
Город	425	411	436
Село	283	283	277
Форма ТБ
Легочная	684	668	683
Внелегочная	23	22	27
Легочная в сочетании с внелегочной	1	4	3
Исход лечения (когорта 2017–2018 гг.)
Вылечен	310	218	
Завершение лечения	207	326	
Переведен	57	40	
Неудача лечения	45	33	
Нарушение режима	8	9	
Смерть	81	68	

В 2017 г. было зарегистрировано 17 700 случаев подтвержденного ТБ, из них 4% составляли пациенты с СД, т.е. 708 пациентов. В 2018 г. отмечается снижение заболеваемости ТБ на 21,7%, и количество больных ТБ составило 14 020 человек. Снизилось также количество пациентов с СД и ТБ с 708 до 694. В 2019 г. количество больных ТБ составило 14 320 человек, из них 713 пациентов имели СД (5%).

Результаты проведенного нами анализа позволяют сделать следующие выводы, представляющие интерес для нашего исследования: высокая распространенность заболеваемости ТБ с сопутствующим диагнозом СД встречается в возрастной категории от 45 до 64 лет. К данной категории относятся 1193 больных (56,1% от общего количества). Возрастная категория старше 65 лет составила 22,7%, люди в возрасте от 25 до 44 лет составляли 19,0%. Средний возраст всех исследуемых пациентов с СД составил 54,7±13,4 года. Данные по возрастным категориям исследуемых показаны на рисунке 2.

**Figure:**
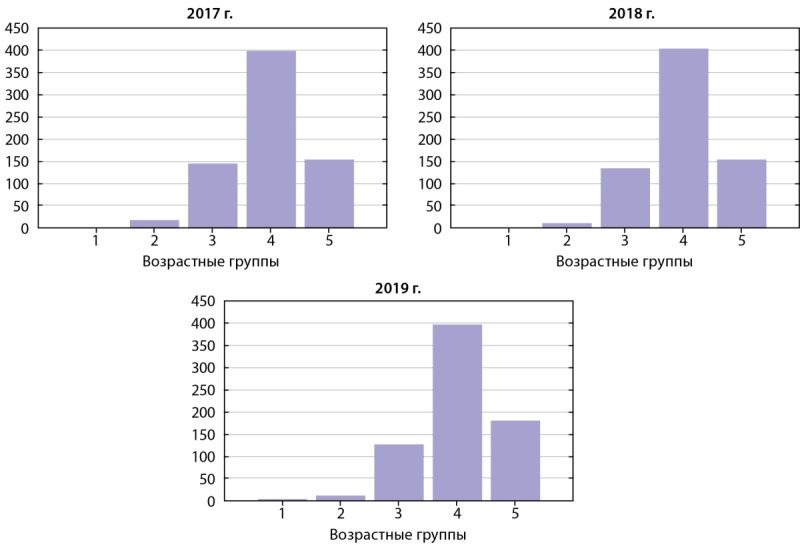
Рисунок 2. Распределение пациентов с туберкулезом и сахарным диабетом по возрастной структуре, категоризированных по годам прохождения лечения (абс. числа).Расшифровка. Возрастные группы: 1 — возрастная группа до 14 лет; 2 — возрастная группа от 15 до 24 лет; 3 — возрастная группа от 25 до 44 лет; 4 — возрастная группа от 45 до 64 лет; 5 — возрастная группа старше 65 лет.

Чаще всего коморбидность СД и ТБ встречалась среди мужчин. В 2017 г. мужчин было 414, а женщин — 294, в следующем, 2018 г.: мужчин — 408, женщин — 286. В 2019 г. аналогичная ситуация сохранялась, мужчин было 424 человека, женщин — 289. Таким образом, мужчин с сочетанным диагнозом ТБ и СД в среднем было на 30,2% выше, чем женщин.

Анализируя данные пациентов, было выявлено, что большее количество заболевших проживали в городе. В 2017 г. пациенты с городским местом жительства опережали по количеству на 31,6% пациентов с сельским местом жительства (город — 425, село — 283), в 2018 г. была отмечена разница в 30,6% (город — 411, село — 283), а в 2019 г. разница составляла 34,6% (город — 436, село — 277).

В изучаемый период у превалирующего большинства пациентов с СД (95,8–96,6%) отмечалась легочная форма ТБ. У остальных пациентов (3,2–3,8%) диагностировалась внелегочная форма ТБ. Сочетанная форма ТБ, т.е. легочная в сочетании с внелегочной, выявлялась редко: в 2017 г. — 1, в 2018 г. — 4 и в 2019 г. — 3 случая.

Конечным этапом исследования было определение исходов лечения у пациентов с СД и ТБ. Изучаемым периодом были 2017 и 2018 гг.

Анализируя результаты 2017 г., хочется отметить низкую эффективность лечения больных с коморбидностью. Вылеченные составляют 43,7% от общего количества диагностированных, из 708 больных с сочетанным диагнозом ТБ и СД 310 вылечились полностью от ТБ. Лечение завершили 207 больных (29,3%), переведенных пациентов было 57 (8,1%). У 6,3% пациентов, т.е. у 45, зарегистрирована неудача лечения, нарушение режима отмечено у 8 (1,1%), летальный исход зафиксирован у 81 (11,4%) пациента.

В 2018 г. больше было пациентов из категории «завершившие лечение», из 694 пациентов — 326 (47,1%). Вылеченных, по сравнению с прошлым годом, было меньше, они составили лишь 31,4% от общего числа пациентов за 2018 г. (218 пациентов). Переведенных пациентов было 40 (5,8%), неудача лечения отмечалась у 33 (4,7%), нарушение режима — 9 (1,3%) больных. В 2018 г. умерли 68 пациентов (9,8%), что на 1,6% ниже по сравнению с 2017 г.

## ОБСУЖДЕНИЕ

Распространенность СД среди больных ТБ в данном исследовании за 2017–2019 гг. в среднем составила 4,6%, т.е. в 2017 г. — 4%, 2018 г. — 5% и 2019 г. — также 5%.

В систематическом обзоре, опубликованном в 2017 г., сообщается, что общая глобальная медианная распространенность СД среди пациентов с ТБ составляет 16% (IQR 9,0–25,3%) [8][9]. Самый низкий показатель был выявлен в Республике Бенин — 1,9%, а самый высокий — на Маршалловых островах (остров Эбай), где распространенность оценивалась до 45% [10][11], вышеуказанные данные за 2014 и 2015 гг.

В азиатских странах общая медианная распространенность СД среди пациентов с ТБ оценивается в 17% (IQR 11,4–25,8%) [12][13]. Таким образом, можно сделать вывод, что в Казахстане уровень коморбидности СД с ТБ держится на уровне ниже среднего.

Во многих исследованиях также регистрировалась более высокая распространенность СД среди мужчин по сравнению с женщинами [11]. В нашем исследовании мужчин было на 30,3% больше (всего: мужчин — 1246, женщин — 869). Одной из причин этого может быть более высокая частота таких привычек, как курение и употребление алкоголя среди мужчин. Тем не менее многомерный анализ не мог показать значительную связь [11].

Основной целью исследования, как упоминалось выше, было выявление связи между исходом и возрастом лечащихся. Было доказано, что возраст является значительным фактором риска, который также отмечается многими другими исследователями [11–15]. Отмечается положительная корреляция между возрастом и исходом лечения пациентов с ТБ. Смертность была выше у представителей возрастных групп старше 45 лет — OR (95% CI) 0,213 (0,019–2,362), p — 0,0000015 (p<0,05). Результаты статистических данных по всем возрастным группам и исходам представлены на рис. 3.

**Figure:**
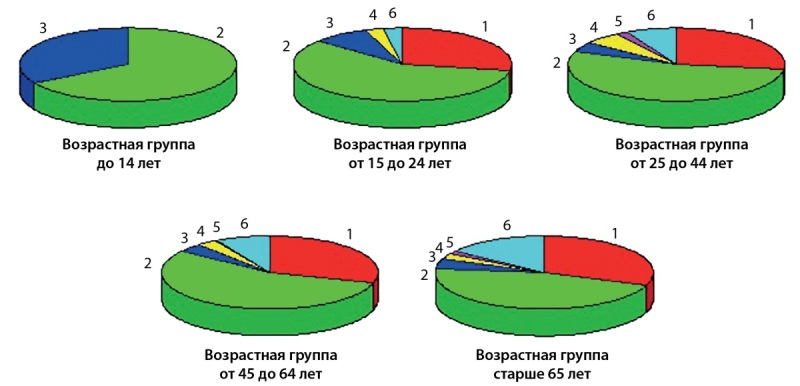
Рисунок 3. Исходы лечения пациентов с СД и ТБ по всем исследуемым возрастным группам (процентные показатели).Расшифровка. По исходам лечения: 1 — вылечен; 2 — лечение завершено; 3 — переведен; 4 — неудача лечения; 5 — нарушение режима; 6 — смерть.

Основной причиной неблагоприятного исхода может являться снижение иммунного статуса, одного из факторов риска как для ТБ, так и для СД. По результатам статистических расчетов сделан вывод, что тип ТБ также может иметь влияние на исход лечения — OR (95% CI) 0,822 (0,404–1,670), p — 0,00001 (p<0,05) (табл. 2).

**Table:** Таблица 2. Влияние на исход возраста и типа ТБ у пациентов

Критерии для оценки корреляции с исходом	Исход
OR (95% CI)	p-value
Возраст	0,213 [ 0,019–2,362]	0,0000015
Локализация ТБ	0,822 [ 0,404–1,670]	0,00001

## ЗАКЛЮЧЕНИЕ

Наше исследование носило несколько ограничений. Во-первых, наблюдение было относительно коротким, особенно для цикла данных по исследованию распространенности СД среди ТБ, по этой причине общая медиана, возможно, будет некорректна. Мы указываем лишь среднее число за последние 3 года (2017–2019 гг.). Во-вторых, в свидетельстве о смерти была зарегистрирована только одна отмеченная причина — СД, хотя стоит учитывать и другие заболевания, такие как заболевания сердечно-сосудистой системы, острые нарушения мозгового кровообращения и т.д.

При этом проведенная работа имеет и сильную сторону — в РК впервые проведен общий анализ распространенности СД среди больных ТБ.

В результате исследования за 2017–2019 гг. был получен материал, анализ которого позволил заключить, что исход лечения в виде смертности прямо пропорционален возрасту пациента. Т.е. у представителей старших возрастных групп вероятность смертности выше, чем у молодых пациентов.

## ДОПОЛНИТЕЛЬНАЯ ИНФОРМАЦИЯ

Источники финансирования. Работа выполнена по инициативе авторов без привлечения финансирования.

Конфликт интересов. Все авторы декларируют отсутствие явных и потенциальных конфликтов интересов, связанных с публикацией настоящей статьи.

Участие авторов. Все авторы одобрили финальную версию статьи перед публикацией, выразили согласие нести ответственность за все аспекты работы, подразумевающую надлежащее изучение и решение вопросов, связанных с точностью или добросовестностью любой части работы.

## References

[cit1] World Health Organization (WHO). Global Tuberculosis Report 2019. Geneva: 2020 [cited 14.04.2020]. Available from: https://apps.who.int/iris/bitstream/handle/10665/274453/9789241565646-eng.pdf?ua=1

[cit2] Davis A., Terlikbayeva A., Aifah A., Hermosilla S., Zhumadilov Z., Berikova E., Rakhimova S., Primbetova S., Darisheva M., Schluger N., El-Bassel N. (2016). Risks for tuberculosis in Kazakhstan: implications for prevention. The International Journal of Tuberculosis and Lung Disease.

[cit3] International Diabetes Federation. Diabetes atlas. IDF. 9th. 2019. [cited 14.04.2020] Available from: https://www.diabetesatlas.org/upload/resources/2019/IDF_Atlas_9th_Edition_2019.pdf

[cit4] Mukasheva Assel, Saparkhojayev Nurbek, Akanov Zhanay, Apon Amy, Kalra Sanjay (2019). Forecasting the Prevalence of Diabetes Mellitus Using Econometric Models. Diabetes Therapy.

[cit5] Ugarte-Gil Cesar, Alisjahbana Bachti, Ronacher Katharina, Riza Anca Lelia, Koesoemadinata Raspati C, Malherbe Stephanus T, Cioboata Ramona, Llontop Juan Carlos, Kleynhans Leanie, Lopez Sonia, Santoso Prayudi, Marius Ciontea, Villaizan Katerine, Ruslami Rovina, Walzl Gerhard, Panduru Nicolae Mircea, Dockrell Hazel M, Hill Philip C, Mc Allister Susan, Pearson Fiona, Moore David A J, Critchley Julia A, van Crevel Reinout (2019). Diabetes Mellitus Among Pulmonary Tuberculosis Patients From 4 Tuberculosis-endemic Countries: The TANDEM Study. Clinical Infectious Diseases.

[cit6] Lee Meng-Rui, Huang Ya-Ping, Kuo Yu-Ting, Luo Chen-Hao, Shih Yun-Ju, Shu Chin-Chung, Wang Jann-Yuan, Ko Jen-Chung, Yu Chong-Jen, Lin Hsien-Ho (2016). Diabetes mellitus and latent tuberculosis infection: a systemic review and meta-analysis. Clinical Infectious Diseases.

[cit7] Alebel Animut, Wondemagegn Amsalu Taye, Tesema Cheru, Kibret Getiye Dejenu, Wagnew Fasil, Petrucka Pammla, Arora Amit, Ayele Amare Demsie, Alemayehu Mulunesh, Eshetie Setegn (2019). Prevalence of diabetes mellitus among tuberculosis patients in Sub-Saharan Africa: a systematic review and meta-analysis of observational studies. BMC Infectious Diseases.

[cit8] Workneh Mahteme Haile, Bjune Gunnar Aksel, Yimer Solomon Abebe (2017). Prevalence and associated factors of tuberculosis and diabetes mellitus comorbidity: A systematic review. PLOS ONE.

[cit9] Sane Schepisi Monica, Navarra Assunta, Altet Gomez M Nieves, Dudnyk Andrii, Dyrhol-Riise Anne Margarita, Esteban Jaime, Giorgetti Pier Francesco, Gualano Gina, Guglielmetti Lorenzo, Heyckendorf Jan, Kaluzhenina Anna, Lange Berit, Lange Christoph, Manika Katerina, Miah Jalal, Nanovic Zorica, Pontali Emanuele, Prego Monica Rios, Solovic Ivan, Tiberi Simon, Palmieri Fabrizio, Girardi Enrico (2018). Burden and Characteristics of the Comorbidity Tuberculosis—Diabetes in Europe: TBnet Prevalence Survey and Case-Control Study. Open Forum Infectious Diseases.

[cit10] Nasa J. N., Brostrom R., Ram S., Kumar A. M. V., Seremai J., Hauma M., Paul I. A., Langidrik J. R. (2014). Screening adult tuberculosis patients for diabetes mellitus in Ebeye, Republic of the Marshall Islands. Public Health Action.

[cit11] Ade S., Affolabi D., Agodokpessi G., Wachinou P., Faïhun F., Toundoh N., Békou W., Makpenon A., Ade G., Anagonou S., Harries A. D. (2015). Low prevalence of diabetes mellitus in patients with tuberculosis in Cotonou, Benin. Public Health Action.

[cit12] Hoa N. B., Phuc P. D., Hien N. T., Hoa V. Q., Thuong P. H., Anh P. T., Nhung N. V. (2018). Prevalence and associated factors of diabetes mellitus among tuberculosis patients in Hanoi, Vietnam. BMC Infectious Diseases.

[cit13] Workneh Mahteme Haile, Bjune Gunnar Aksel, Yimer Solomon Abebe (2016). Prevalence and Associated Factors of Diabetes Mellitus among Tuberculosis Patients in South-Eastern Amhara Region, Ethiopia: A Cross Sectional Study. PLOS ONE.

[cit14] Ncube R. T., Dube S. A., Machekera S. M., Timire C., Zishiri C., Charambira K., Mapuranga T., Duri C., Sandy C., Dlodlo R. A., Lin Y. (2019). Feasibility and yield of screening for diabetes mellitus among tuberculosis patients in Harare, Zimbabwe. Public Health Action.

[cit15] Song Wan-mei, Shao Yang, Liu Jin-yue, Tao Ning-ning, Liu Yao, Zhang Qian-yun, Xu Ting-ting, Li Shi-jin, Yu Chun-bao, Gao Lei, Cui Liang-liang, Li Yi-fan, Li Huai-chen (2019). <p>Primary drug resistance among tuberculosis patients with diabetes mellitus: a retrospective study among 7223 cases in China</p>. Infection and Drug Resistance.

